# Realizing quinary charge states of solitary defects in two-dimensional intermetallic semiconductor

**DOI:** 10.1093/nsr/nwab070

**Published:** 2021-04-24

**Authors:** Jian Gou, Bingyu Xia, Xuguang Wang, Peng Cheng, Andrew Thye Shen Wee, Wenhui Duan, Yong Xu, Kehui Wu, Lan Chen

**Affiliations:** Institute of Physics, Chinese Academy of Sciences, Beijing 100190, China; School of Physics, University of Chinese Academy of Sciences, Beijing 100049, China; Department of Physics, National University of Singapore, Singapore117542, Singapore; State Key Laboratory of Low-Dimensional Quantum Physics, Department of Physics, Tsinghua University, Beijing 100084, China; Institute of Physics, Chinese Academy of Sciences, Beijing 100190, China; School of Physics, University of Chinese Academy of Sciences, Beijing 100049, China; Institute of Physics, Chinese Academy of Sciences, Beijing 100190, China; School of Physics, University of Chinese Academy of Sciences, Beijing 100049, China; Department of Physics, National University of Singapore, Singapore117542, Singapore; State Key Laboratory of Low-Dimensional Quantum Physics, Department of Physics, Tsinghua University, Beijing 100084, China; Institute for Advanced Study, Tsinghua University, Beijing 100084, China; Collaborative Innovation Center of Quantum Matter, Beijing 100871, China; State Key Laboratory of Low-Dimensional Quantum Physics, Department of Physics, Tsinghua University, Beijing 100084, China; Collaborative Innovation Center of Quantum Matter, Beijing 100871, China; RIKEN Center for Emergent Matter Science (CEMS), Wako, Saitama 351-0198, Japan; Institute of Physics, Chinese Academy of Sciences, Beijing 100190, China; School of Physics, University of Chinese Academy of Sciences, Beijing 100049, China; Songshan Lake Materials Laboratory, Dongguan 523808, China; Institute of Physics, Chinese Academy of Sciences, Beijing 100190, China; School of Physics, University of Chinese Academy of Sciences, Beijing 100049, China; Songshan Lake Materials Laboratory, Dongguan 523808, China

**Keywords:** 2D materials, intermetallic semiconductor, tip-induced band bending, solitary defect, defect charging

## Abstract

Creating and manipulating multiple charge states of solitary defects in semiconductors is of essential importance for solitary defect electronics, but is fundamentally limited by Coulomb's law. Achieving this objective is challenging, due to the conflicting requirements of the localization necessary for the sizable band gap and delocalization necessary for a low charging energy. Here, using scanning tunneling microscopy/spectroscopy experiments and first-principles calculations, we realized exotic quinary charge states of solitary defects in two-dimensional intermetallic semiconductor Sn_2_Bi. We also observed an ultralow defect charging energy that increases sublinearly with charge number rather than displaying the usual quadratic behavior. Our work suggests a promising route for constructing multiple defect-charge states by designing intermetallic semiconductors, and opens new opportunities for developing quantum devices with charge-based quantum states.

## Teaser

Quinary charge states of solitary defects are realized and manipulated in 2D intermetallic semiconductor, displaying unusually delocalized defect states and ultralow charging energy, suggesting possible application in charge-based qubits.

## INTRODUCTION

A single-atom defect is the smallest structural unit of a solid material. The construction of devices based on a single defect can reach the limit of miniaturization of semiconductor devices. The peculiar electronic characteristics of the single defect make it solitarily distinct from its surroundings for information recording and processing. In past decades, the creation and manipulation of single defects in semiconductors has led to the development of a new research field called solitary defect electronics (optoelectronics) or solotronics [1,2]. The detection of discrete characteristics of solitary defects in semiconductors has motivated new device designs in which solitary defects can be used to physically realize ‘qubits’ of solid-state quantum computation through electron spin [3,4] or charge [5]. Solitary defects have also been proposed as non-classical light sources in quantum information sciences including quantum key distribution systems [6], quantum repeaters [7] and local sensors [8]. Generally, charge and spin are the two fundamental degrees of freedom in solitary defects. Due to the low-energy multiple states of spin momentum, solotronics research has mostly focused on the studies of spin quantum computing [3,4,9–11]. However, in contrast to the use of optical signals and magnetic fields for spin manipulation [12–18], multiple charge states can be written and read through the application of an electric field, enabling a more compact device size and compatibility with modern electronics technology [19]. Thus, realization of tunable multiple charge states in solitary defects can provide an important alternative avenue to quantum devices, and is highly desirable.

For a successful charging process, the injection of a single charge must overcome the Coulomb repulsion energy (*U*) while the defect state must be kept within the band gap. However, successive multiple charging processes increase the charging energy (*ΔE*) quadratically (*ΔE* = *UN^2^/2*, where *N* is the charge number) [20,21], easily exceeding the band gap and leading to charging failure. Therefore, only one or two charging processes have been observed in previous work [22–33]. To realize multiple (particularly more than two) charge states in solitary defects, a wide band gap and delocalized defect states corresponding to small *U* values are required. However, these two material requirements are contradictory because a wide band gap is usually associated with strong localization. Thus, finding a suitable compromise between the band gap and delocalization is a key challenge.

**Figure 1.**
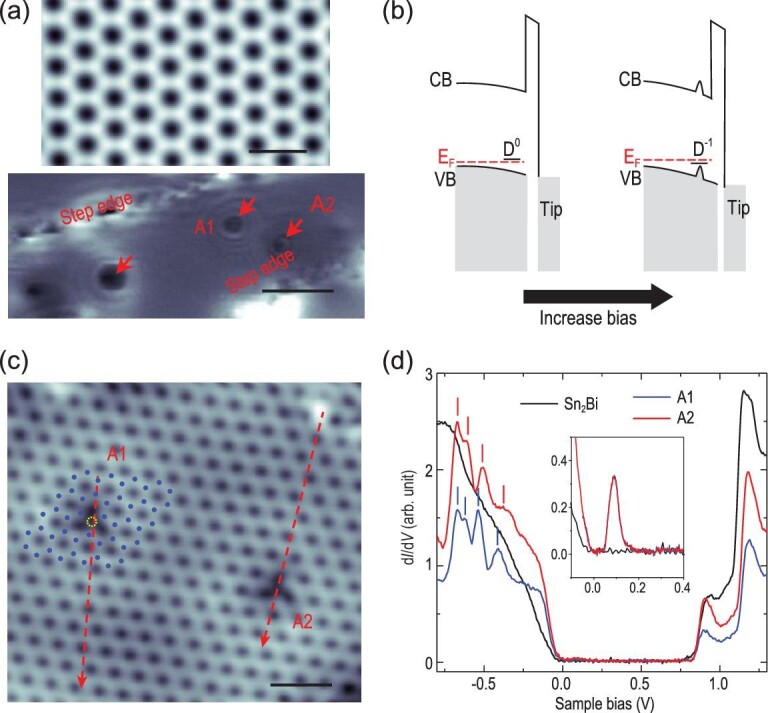
Solitary defects in Sn_2_Bi/Si(111). (a) Upper panel: typical high-resolution STM image of Sn_2_Bi (setpoint: *V_S_* = −1.0 V, *I *= 90 pA; scale bar, 1.4 nm). Lower panel: differential conductance (d*I*/d*V*) map (setpoint: *V_S_* = −0.6 V, *I *= 290 pA; scale bar, 9.0 nm) of a large area with solitary defects surrounded by several rings (indicated by red arrows). (b) Schematics of the tip-induced acceptor charging process for one electron. (c) STM image of Sn_2_Bi with two defects: A1 and A2 (setpoint: *V_S_* = 1.0 V, *I* = 400 pA; scale bar, 2.0 nm). The locations of top Bi atoms near A1 are highlighted by blue dots. The yellow dotted circle labels the position of A1. (d) d*I*/d*V* spectra obtained on defects A1 (blue), A2 (red) and bare surface of Sn_2_Bi (black) (initial setpoint: *V_S_* = −1.0 V, *I* = 400 pA). Inset indicates the related STS measurements of the in-gap state (initial setpoint: *V_S_* = −0.2 V, *I* = 90 pA). Vertical red (blue) bars highlight four peaks in the occupied states of A2 (A1).

The use of intermetallic semiconductors with delocalized in-gap defect states is a promising solution for the problem of the trade-off between the band gap and delocalization. Materials in the two-dimensional (2D) limit can have various chemical stoichiometries and atomic arrangements, thus offering numerous opportunities to overcome the challenge posed by the trade-off between the band gap and delocalization. Recently, a 2D semiconductor Sn_2_Bi was synthesized on the (111) silicon surface, and was found to have an indirect band gap of ∼0.8 eV [34]. A typical atomic scanning tunneling microscopy (STM) image and structure models of this system are shown in Fig. 1(a) and Fig. 4(b) and (c), respectively. A unique honeycomb lattice is formed by the topmost Bi atoms, the sublattices of which are connected indirectly with each other by bonding with Sn tetramers.

## RESULTS

In this 2D material, STM measurements observed one special type of defect surrounded by several concentric rings in the differential conductance (d*I*/d*V*) map (Fig. 1(a)). The surrounding ring in the d*I*/d*V* map can be ascribed to a charging process of solitary defects caused by tip-induced band bending (TIBB) [22–33], as schematically illustrated in Fig. 1(b). Specifically, the electric field between the tip and the sample induces surface band bending, leading to a shift of the in-gap defect states with respect to the Fermi level. The shift changes gradually with varying bias voltage or tip-defect distance. Once they are aligned with the Fermi level, the defect states will be charged and open an additional tunneling channel, giving rise to a peak observed in scanning tunneling spectroscopy (STS) and a surrounding ring in the d*I*/d*V* map. Similar phenomena have been observed in the STM experiments on the semiconductor surfaces such as Si(111) [23], GaAs(110) [24], (Sr_1−x_La)_2_IrO_4_ [27] and ZnO(0001) [28,29], and several 2D systems/thin films such as single-layer C_60_ film [30], Bi_2_Se_3_ [22], graphene/SiO_2_ [25] and graphene/*h*-BN [26]. However, based on the fact that one (two) surrounding ring(s) corresponds (correspond) to one (two) charging process(es) of the defect, several rings should be the result of multiple successive charging processes that have not been reported in previous work.

**Figure 2.**
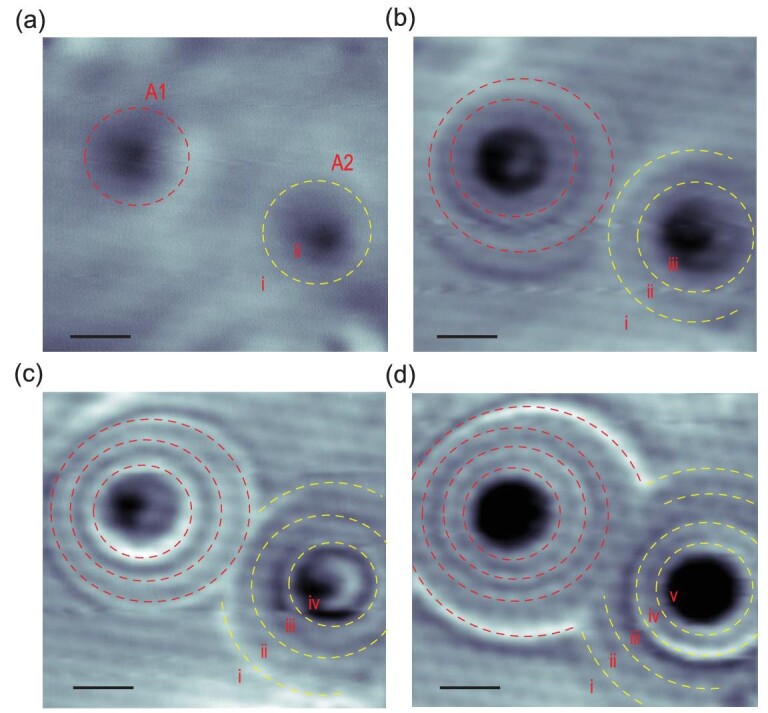
Multiple charging processes of solitary defects in Sn_2_Bi/Si(111). Differential conductance maps of A1 and A2 taken at a series of energies (setpoint: (a) *V_S_* = −0.35 V, *I* = 300 pA; (b) *V_S_* = −0.5 V, *I* = 400 pA; (c) *V_S_* = −0.6 V, *I* = 250 pA; (d) *V_S_* = −0.75 V, *I* = 400 pA; scale bars, 2.0 nm). Both the number and diameter of the charging rings increase as the voltage rises gradually. Red (yellow) dashed circles/arcs are marked in the figure to highlight the rings of A1 (A2). The labels (i, ii, iii, iv and v) represent different defect charge states.

To thoroughly understand the multiple charging processes, we first investigated the atomic structure and the local density of states (LDOS) of the defect. Figure 1(c) shows the atomic-resolution STM image containing two defects (A1 and A2). The d*I*/d*V* curves obtained on A1 and A2, as well as on the bare Sn_2_Bi are shown in Fig. 1(d). Compared to bare Sn_2_Bi, an in-gap defect state above the Fermi level and four peaks in the range of −0.35 eV to −0.7 eV with respect to the Fermi level are observed in the STS spectra of the defects. The closed examination indicates there are four sub-peaks in the envelope of in-gap state (Fig. S2(a)), indicating there are four intrinsic, nearly degenerated defect levels in single defects. These four STS peaks correspond to four charging processes caused by the varying bias voltage, implying that four electrons are successively injected into the defect states and quinary defect charge states are realized. Moreover, we measured a series of d*I*/d*V* maps in the area containing defects A1 and A2 with certain bias voltages and varying tip-defect distance, as shown in Fig. 2. It is found that in the range between −0.35 eV and −0.7 eV with respect to the Fermi level, the number of rings increases from one to four, corresponding to the charge states varying from two (i and ii) to five (i, ii, iii, iv and v). The outermost ring corresponds to the first charging process and its diameter increases with bias voltage, and successive charging processes occur from outer to inner rings. Since charging occurs at a certain electric field, the distance between the tip and the defect should be larger at higher bias voltage, corresponding to the diameter of the rings increasing with the bias voltage in the d*I*/d*V* maps.

**Figure 3.**
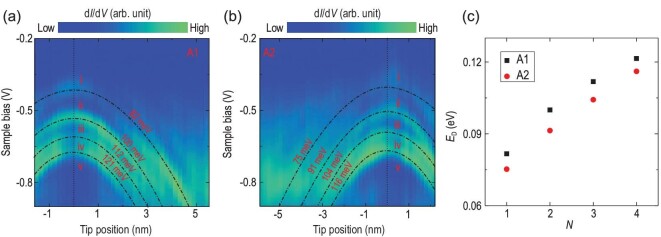
Dependence of charging energy on charge number. Color-coded d*I*/d*V* line mapping over defects (a) A1 and (b) A2 along the red dashed arrows in Fig. 1(c). Initial setpoint: *V_S_* = −1.0 V, *I* = 500 pA. Vertical dotted lines indicate the center positions of A1 and A2. The dash-dotted lines represent the theoretical fitting of the position-dependent tip-induced band bending (TIBB) at each charging peak. The TIBB energies are also marked. (c) TIBB-derived charging energies of A1 and A2 for four charging peaks vary with charge number *N* (1–4).

To further identify the TIBB-induced multiple charging procedure and quantify the charging energy, we performed STS measurements along the red dashed lines across A1 and A2 in Fig. 1(c) in order to elucidate the dependence of the charging peaks on both bias voltage and tip-defect distance. As shown in Fig. 3(a) and (b), the four charging peaks in occupied states gradually shift to higher binding energies as the tip moves away from the defects, displaying a set of nearly parabolic patterns in the colored line maps. On the other hand, we numerically studied the TIBB model by solving Poisson's equation [35], and obtained the TIBB energies of each charging peak by fitting the nearly parabolic patterns (see details in the Supplementary Data). The results of the model calculations are in good agreement with the experimental data.

The TIBB energies of the four peaks are 82, 100, 112 and 121 meV for A1, and 75, 91, 104 and 116 meV for A2, corresponding to the charging energies of the defect states occupied by 1–4 electrons, as shown in Fig. 3(c). For localized defect states, typically *ΔE = UN^2^/2* and *U* is on the order of eV (e.g. 1.2 eV for the dangling bond of the Si surface [20,21]). By contrast, the charge energies of the present system are ∼0.1 eV, approximately one order of magnitude smaller than usual. More importantly, the increase in the charging energy with increasing charge number is not quadratic but sublinear, much slower than normally expected. The ultralow defect charging energy and the sublinear dependence behavior are quantitatively and even qualitatively distinct from the typical quadratic charging behaviors of defects.

## DISCUSSION

**Figure 4.**
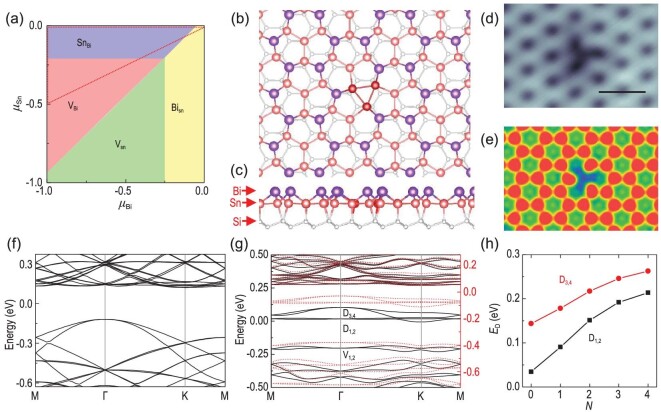
First-principles study of solitary defects in Sn_2_Bi/Si(111). (a) Phase diagram of intrinsic surface defects in Sn_2_Bi for varying atomic chemical potentials (*μ*_Sn_ and *μ*_Bi_). The red dotted triangle denotes Sn-rich and Bi-poor conditions, corresponding to the experimental growth conditions that favor the formation of bismuth vacancy (V_Bi_) or tin antisite (Sn_Bi_). (b and c) Top and side views of the atomic structure of Bi vacancy in Sn_2_Bi/Si(111). Sn trimer at the Bi vacancy is colored deep red for clarity. (d) Experimental (setpoint: *V_S_* = 1.0 V, *I* = 400 pA; scale bar, 1.3 nm) and (e) simulated STM images of Bi vacancy. Scale bar, 1.3 nm. (f and g) Band structures of the 8 × 8 surface supercell (f) without and (g) with one Bi vacancy. In (g), black solid and red dotted lines represent the band structures of the defect charge states *N* = 1 and 4, respectively. (h) Defect energy levels of D_1,2_ and D_3,4_ (referenced to the valence band maximum of Sn_2_Bi) for different defect charge states. *N* denotes the occupation of the in-gap defect states.

To understand the charging behaviors of the in-gap defect states, we performed first-principles calculations on the atomic defects of 2D Sn_2_Bi on Si(111). We studied various types of intrinsic defects including vacancies, adatoms and antisites (see details in the Supplementary Data), and calculated the defect formation energies as a function of the atomic chemical potentials, obtaining the surface defects phase diagram presented in Fig. 4(a). Our results suggest that under the experimental Bi-poor and Sn-rich conditions [33], the defect with the lowest formation energy is either the bismuth vacancy (V_Bi_) or tin antisite (Sn_Bi_: Sn on a Bi site). The calculated band structures (Fig. 4(g) and Fig. S4) reveal that V_Bi_ and Sn_Bi_ are p-type dopants with three and one charge carriers per defect, respectively. However, four in-gap defect levels (D_1,2_ and D_3,4_) are found for V_Bi_, but none for Sn_Bi_. Thus, the former type of defect is highly likely to be the experimentally observed defect. Moreover, the defects show a dark triangular star shape in the STM image (Fig. 1(c)). According to the well-established structure of Sn_2_Bi [34], the bright honeycomb lattice in the STM image corresponds to the bismuth atoms, while the defect located at the corner of a honeycomb is ascribed to the absence of one Bi atom. The STM image is reproduced well by the theoretical simulation of V_Bi_ (Fig. 4(d) and (e)). Therefore, V_Bi_ is identified as the native solitary defect observed in our experiment.

The atomic structure of V_Bi_ is shown in Fig. 4(b) and (c), where one Bi atom located above the H_3_ site of Si(111) is absent, leaving three dangling bonds on the three neighboring Sn atoms. While the multiple dangling bonds are usually eliminated by structural reconstruction, they are stable in Sn_2_Bi because the atomic framework of 2D Sn_2_Bi is fixed by the Si(111) substrate, making structural reconstruction energetically unfavorable. The total energy of this system is lowered by surface structural relaxation that preserves the C_3_ rotational symmetry as observed by STM. Around the Bi vacancy, the length of the Sn−Bi bonds increases slightly by 0.06 Å, while the distance between the neighboring Sn atoms decreases significantly from 4.17 Å to 3.29 Å, leading to the formation of an Sn trimer.

Figure 4(f) and (g) displays the band structures of Sn_2_Bi with and without a Bi vacancy in an 8 × 8 Si(111) surface supercell, respectively. Comparison of these two band structures enables us to identify the four in-gap defect states (D_1,2_ and D_3,4_ at the Γ point) contributed by V_Bi_, which accord with the experimental observation of four in-gap defect levels in STS (inset of Fig. 1(d) and Fig. S2) very well. Intrinsically, since only neutral charge carriers are considered in the calculations, one electron already occupies D_1,2_, leading to a slight deviation from the experimentally observed four unoccupied defect states prior to the application of the tip. Keeping this in mind, we theoretically simulated multiple charge states of V_Bi_ by adding a different number of electrons (*ΔN* = −1, 0, 1, 2, 3) into the supercell. As shown in Fig. 4(g) and Fig. S7, all of the added electrons are filled into the in-gap defect states with the total charge number of *N = ΔN + *1. The charging process has a minor influence on the band dispersion except for the in-gap defect states that shift upwards with respect to the other bands. While the observed shift increases with increasing electron charge, the defect states always stay within the band gap. Thus, V_Bi_ can display quinary charging states labeled by *N* = 0, 1, 2, 3, 4. As displayed in Fig. 4(h), the calculated upward shifts of D_1,2_ and D_3,4_ defect levels increase with increasing *N* due to the enhanced Coulomb repulsion arising from more charges. Noticeably, the increase of energy is quite slow in this case and becomes even slower when charging-induced structural relaxation is considered (see details in the Supplementary Data), suggesting an ultralow defect charging energy, in agreement with our experimental observations. It is noted that the TIBB-derived charging energies should correspond to the sum of the Coulomb repulsion energy between the defect charges, and the energy difference between the defect levels. The STS experiments indicate that the total energy difference between the lowest and highest defect levels is as small as 60 meV (Fig. S2(a) and (b)), suggesting that the TIBB-derived charging energies are mainly due to the Coulomb repulsion energy. The results of the calculations indicate that the Coulomb repulsion between the defect charges is quite low, in qualitative agreement with our experiments.

**Figure 5.**
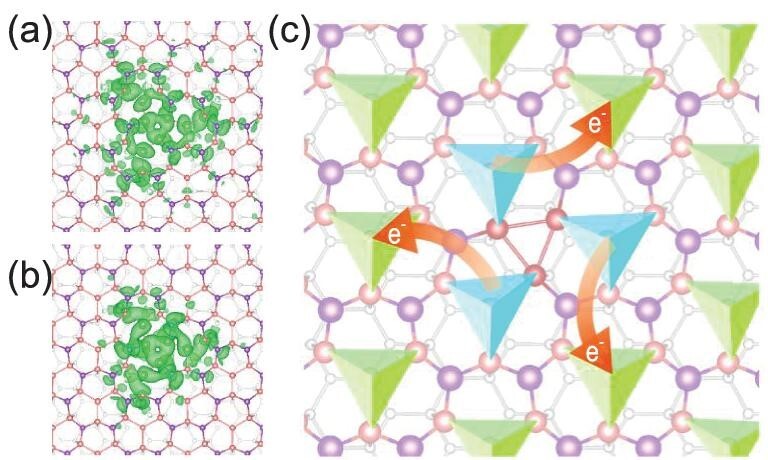
Defect states in Sn_2_Bi. (a and b) Isosurfaces of the charge density for defect states (a) D_1,2_ and (b) D_3,4_. (c) Schematic diagram showing the distribution of defect charge. In 2D Sn_2_Bi, Sn tetramers are separated from each other by Bi atoms. Around the Bi vacancy, three Sn tetramers (denoted by blue pyramids) are connected by an Sn trimer (colored red), providing ample space to host the defect charges. Upon the addition of more charges into the defect, the added charges (*e^–^*) will flow across the Bi atoms and spread out to the neighboring Sn tetramers (denoted by green pyramids), leading to a more delocalized defect-charge distribution.

Detailed theoretical analysis of the electronic states of V_Bi_ revealed several important features. As shown in Fig. 4(g), defect states D_1,2_ appear as flat bands that are expected for localized dangling bonds, whereas D_3,4_ show significant band widths (∼0.11 eV) despite the quite large supercell (30.7 Å × 30.7 Å), implying their unusually delocalized nature. Figure 5(a) and (b) shows the real-space distributions of D_1,2_ and D_3,4_, respectively, that are not simply localized near V_Bi_, but rather are delocalized among the three neighboring Sn tetramers. In turn, the delocalized distribution results in a low defect charge concentration and weak Coulomb repulsion give rise to the ultralow defect charging energy. Moreover, our calculations find that the defect charge distribution becomes more delocalized with greater defect charges (Fig. S8). This rationalizes the experimental finding of the sublinear growth of the charging energy.

The extraordinarily delocalized defect states are inherently related to the two advantages of Sn_2_Bi, namely its unique atomic structure and chemical composition. As schematically depicted in Fig. 5(c), 2D Sn_2_Bi is composed of periodic Sn tetramers separated by Bi atoms. When a Bi vacancy is created, the three neighboring Sn tetramers bond to each other, providing adequate space for hosting the defect charges. Moreover, Sn_2_Bi is a binary semiconductor formed by two special elements Sn and Bi. In their elemental states, Sn and Bi are metals and their electronegativities are very close to each other (1.96 for Sn and 2.02 for Bi on the Pauling scale) [36], facilitating charge hopping between Sn and Bi. Thus, the added defect charges can easily flow across Bi atoms and spread out to the next nearest Sn tetramers, making the defect charge distribution even more delocalized, as sketched in Fig. 5(c).

We next discuss the possible guiding principles for the realization of multiple charge states of an in-gap solitary defect. Firstly and obviously, the multiple charging process requires multiple defect states in solitary defects. Secondly and more importantly, the candidate materials are required to satisfy the conditions of a sizable band gap and weak Coulomb repulsion. However, the former condition implies localized electronic states, while the latter requires delocalization. The two conflicting requirements usually cannot be satisfied simultaneously. Previous works reported the observation of two charging processes on the ZnO(0001) surface with interstitial Zn atoms [28,29] where the relatively large band gap of ZnO (over 3.0 eV) enables the double charging of the defect states while still keeping the energy of the defect states within the band gap. To further increase the number of in-gap charge states, in addition to a suitable band gap, it is also necessary to obtain favorable defect properties. Accordingly, our work points out a promising solution to this challenging problem, namely the design of intermetallic semiconductors with network structures and heavy elements near metalloids (e.g. In, Sn, Pb, Sb, Bi) by analogy to Sn_2_Bi. The network configuration helps generate the long-range influence of the defects. Heavy elements near metalloids have metallic elemental forms and show similar electronegativities so that their semiconducting compounds can achieve defect states that are much more delocalized than in the usual semiconductor materials. Theoretically studying the defect charging effects of 2D Sn_2_X (X = Bi, Sb, As, P), we find that the Coulomb repulsion of defect charges decreases along with the decrease in the difference between the electronegativities of the constituent elements (Fig. S10).

Following the above design principle, new intermetallic semiconductors with properties comparable to, or even superior to, those of 2D Sn_2_Bi may be developed using material databases and big-data methods. The search for intermetallic semiconductors with multiple, controllable defect charge states may find promising applications in solitary defect electronics and optoelectronics for quantum computing and communication, and open new opportunities for future research.

## METHODS

The methods are available in the supplementary materials.

## Supplementary Material

nwab070_Supplemental_File
