# Supplementary material for: Realizing quinary charge states of solitary defects in two-dimensional intermetallic semiconductor
Source: Natl Sci Rev. 2021 Apr 24;9(2):nwab070. doi: 10.1093/nsr/nwab070 (PMC8881213; doi:10.1093/nsr/nwab070)
Supplement: nwab070_Supplemental_File [file nwab070_supplemental_file.docx]

Supplementary Materials for

**Realizing Quinary Charge States of Solitary Defects in Two-Dimensional Intermetallic Semiconductor**

Jian Gou, Bingyu Xia, Xuguang Wang, Peng Cheng, Andrew Thye Shen Wee, Wenhui Duan, Yong Xu, Kehui Wu, Lan Chen

**This file includes:**

Experimental methods

Methodology for first-principles calculations

Simulation of tip-induced band bending (TIBB).

In-gap defect states.

Discussion of other possibilities that can produce similar multiple rings.

Discussion of the similarity between the Coulomb blockade model and our explanation

First-principles study of the intrinsic defects in 2D Sn_2_Bi

Fig. S1. Calculated TIBB contour dependent on the tip potential energy (*P_T_*) and tip-defect position.

Fig. S2. Closely measurement of in-gap states.

Fig. S3. Different atomic sites in 2D Sn_2_Bi.

Fig. S4. Electronic structures of V_Bi_ and Sn_Bi_ in 2D Sn_2_Bi.

Fig. S5. Test calculations by the DFT-HSE06 method.

Fig. S6. Tests of theoretical methods for charging simulation.

Fig. S7. Electronic structures of Bi vacancy in 2D Sn_2_Bi.

Fig. S8. Real-space charge redistribution induced by successive charging.

Fig. S9. Influence of charging-induced structural relaxation on the electronic structure and defect energy levels of the Bi vacancy in 2D Sn_2_Bi.

Fig. S10. Calculated defect energy levels of X vacancy in 2D Sn_2_X (X = Bi, Sb, As, and P).

References (34, 38-52)

Supplementary Text

**Experimental Methods**

Sn_2_Bi on a Si(111) substrate was prepared by molecular beam epitaxy (MBE) in a home-built MBE-STM union system. Prior to the growth of Sn_2_Bi, the Si(111) substrate with a resistivity of 0.01-0.02 Ω cm was degassed and flashed to approximately 1250 °C to obtain a clean (7×7) reconstruction in a UHV chamber. Sn_2_Bi was synthesized in two steps [34]. First, the β-√3×√3-Bi reconstruction on Si(111) surface was induced by the deposition of approximately 1.2 monolayer Bi followed by annealing at 670 K [39]. Then, Sn was deposited on the β-√3×√3-Bi/Si(111) surface at 470 K to obtain Sn_2_Bi. After deposition, the sample was *in situ* transferred into an STM chamber and STM measurements were performed under a liquid-helium temperature. The STM tip is made of a tungsten wire after chemical etching. Prior to the measurements, the tip was prepared by a V-pulse and Z-pulse on a clean Au(111) surface. The symmetric shape of the tip was checked by scanning a cluster created by a Z-pulse and the cleanness of the tip was confirmed by reproducing the surface states of Au(111) in STS measurements. The STS was measured with a lock-in amplifier using a 10 mV_rms_, 676 Hz sinusoidal modulation superimposed on the bias. All of the STM images were low-pass filtered and were processed using the WSxM software [40].

**Methodology for first-principles calculations**

Density functional theory (DFT) calculations were performed using the Vienna *ab initio* simulation package (VASP) [41] using the Perdew-Burke-Ernzerhof (PBE) exchange-correlation functional [42], the projector-augmented-wave ion core potential and the plane wave basis with an energy cutoff of 250 eV. Some computational results were further checked with the all-electron full-potential code FHI-aims [43] using the “light” basis. The DFT-PBE results obtained from VASP were verified by using the Heyd-Scuseria-Ernzerhof (HSE06) hybrid functional [44] as implemented in FHI-aims (Fig. S5). The periodic slab model together with a vacuum layer of 15 Å was employed to simulate Sn_2_Bi on Si(111). The defect states are simulated by the supercell approach, where the band dispersion of the defects states is artificially created by the interactions between the defect and its images. To study solitary defects, a single defect was introduced into a large surface supercell to avoid defect-defect interactions. Systems of different sizes were considered: an *n*×*n* surface supercell of Si(111) (*n* = 6, 8) with the substrate modeled by an *m*-bilayer Si (*m* = 0, 1, 3) saturated by hydrogen atoms on the bottom, called “*n*×*n* supercell with *m*-bilayer Si” for simplicity. *m* = 0 means that the Si(111) substrate was replaced by hydrogen atoms in DFT calculations, for which the calculated band structure of Sn_2_Bi is basically the same as that for *m* = 1, 3. Therefore, (*n* = 6, *m* = 0) were selected for the computationally expensive DFT-HSE06 calculations, whereas (*n* = 6, *m* = 3) and (*n* = 8, *m* = 1) were selected for the DFT-PBE calculations of defect formation energies and defect charging states, respectively. The 1×1×1 and 3×3×1 Monkhorst-Pack *k*-point meshes were used for structural relaxation and self-consistent electronic structure calculations, respectively. A Gaussian smearing of 0.1 eV and a force convergence criterion of 0.01 eV/Å were applied, and the spin-orbit coupling (SOC) was included in the self-consistent calculations. The STM image was simulated by calculating the local density of states using the Tersoff-Hamann approach [45]. The defect formation energy was defined as *E*_formation_ = *E*_defect_ – *E*_intrinsic_ – ∑*n*_X_*μ*_X_, where *E*_defect_ and *E*_intrinsic_ represent total energies of the supercell with and without defects, respectively. The element is labelled by X = Sn, Bi. *n*_X_ is the number of atoms added (*n*_X_ > 0) or removed (*n*_X_ < 0) to create an intrinsic defect in the supercell. *μ*_X_ is the atomic chemical potential that was given in reference to the bulk energy of its elementary substance.

Excess charges were introduced into DFT simulation by two approaches: (i) In VASP calculations, a homogeneous background of compensating charge was added to make the whole system charge neutral and to avoid diverging Hartree energy; (ii) in FHI-aims calculations, the compensating charge was also introduced, but by applying the virtual crystal approximation [46, 47] through modifying the nuclear charge of Sn and Bi atoms equally by a small amount as was done in previous work [48]. The two approaches were verified to give consistent results (Fig. S6), and results obtained using the first approach were presented. The deep energy level of Si atoms that is located approximately 8.5 eV below the valence band maximum (VBM) of Sn_2_Bi was used to align the band structures of different charging states. Other deep energies of Si were also chosen for the purpose, and give essentially the same results. The defect energy levels were referenced to the VBM of Sn_2_Bi. In DFT calculations, structural relaxations were performed without considering the charging effects by default. The charging effects can modify the optimized atomic structures slightly and their influence on the electronic structures and defect energy levels were checked (Figs. S9). The energy zero refers to the Fermi energy in all of the calculated band structures.

**Simulation of tip-induced band bending (TIBB).**

The tip-induced band bending (TIBB) is related to the electric field between the tip and the substrate. The extent of TIBB on defects is determined by the tip potential energy and tip-defect distance. In our experiment, the bulk acceptor concentration [49] is approximately 1.2×10^19^ cm^-3^ and the tip-sample distance is estimated to be 0.6 nm by controllable tip contact to the sample surface. The potential energy of STM tip can be written as *P_T_* = *eV_S_* + *W_T_* - *W_S_*, where *e* = -1.6 × 10^-19^ C and *V_S_*, *W_T_*, *W_S_* represent the sample bias, and the work functions of the tip and sample, respectively. Thus, the contact potential (*W_T_* - *W_S_*) between the tip and the sample actually enters the bias value of the flatband condition *P_T_* = 0 eV that is related to the materials of the tip and the apex shape [50, 51]. Based on this boundary condition, we solved the Poisson’s equation directly using typical tip parameters (radius = 10 nm, shank angle = 90°) and Feenstra’s code [35], and obtained the TIBB that depends on the tip potential energy and tip-defect distance, as shown in Fig. S1. The calculated value of TIBB can be compared with the experimental STS line mapping of both defects 1 (A1, Fig. 3(a)) and 2 (A2, Fig. 3(b)) and shows excellent agreement with the experimental results. The flatband conditions (*eV_S_* + *W_T_* - *W_S_* = 0) were derived as *V_S_* = -80 mV and *V_S_* = -50 mV, respectively. Our results indicate that the work functions of Sn_2_Bi and STM tip are comparable, implying that the sample bias of the flatband condition (*P_T_* = 0 eV, TIBB = 0 eV) is nearly at the Fermi level.

**In-gap defect states.**

The in-gap defect states can be resolved in the STS spectra shown in the inset of Fig. 1(d) of the main text. A close examination shows that there are four sub-peaks in the envelope of the in-gap peaks obtained by Gaussian fitting [52], as shown in Fig. S2(a). Since the measured spectrum contains the energy shift induced by TIBB, we use the TIBB calculations above to exclude this kind of shift and obtain the accurate positions of the in-gap states as shown in Fig. S2(b). Comparison to the charging energy derived by TIBB simulation in Fig. 3(a) shows that the two sets of results match very well, verifying the correctness of the TIBB calculations.

The delocalized defect states in the band gap can be measured by d*I*/d*V* mapping. In our work, we use the d*I*/d*V* line maps crossing over the defect to inspect the spatial distribution of the defect states. As shown in Fig. S2(c), the size of ~2.3 nm verified the delocalization of the defect states. In DFT calculations, the charge density distribution of the defect states displays a slow decay away from the vacancy and remains observable more than 1 nm away from the center (Figs. 5(a) and 5(b)). The defect states D_3,4_ show noticeable band widths (~0.11 eV) despite the quite large supercell (3.1 nm ×3.1 nm) (Fig. 4(e)). The theoretical length scale (1~3 nm) is comparable to the experimental value of 2.3 nm.

**Discussion of other possible effects that can produce similar multiple rings.**

The multiple rings induced by electron scattering can be excluded by a tip-height dependent d*I*/d*V* study. Here we adjust the tip height above the Bi vacancy by changing the initial setpoint prior to starting a d*I*/d*V* measurement. One set of the obtained data are shown in Fig. S2(d) where the bias of the set point for C2 (-1.3 V) is larger than that of C1 (-1.0 V), corresponding to a larger tip height of C2 compared to C1. Redshifts of the charging peaks in C2 are clearly observed that can be explained by the fact that a larger tip height requires a larger (absolute) voltage to compensate the reduced electric field. If the rings surrounding the defect originate from electron scattering, the position of the peaks will not change when varying the tip height.

By closely checking the d*I*/d*V* maps (Fig. 2), the possibility of electron scattering induced concentric rings around the defect can be excluded further. First, if the concentric rings are the standing wave due to electron scattering, the distance between the neighbor rings should be the same, because the wave number *q* = *2*π*/λ* (λ is the wave length of standing wave) is unique at a certain energy. However, in our work, the distance between the neighbor rings clearly varies (Fig. 2(d)). Second, even though the standing wave around a single point barrier stems from electron scattering and appears similar to the rings surrounding the point, in the presence of two point barriers nearby, the waves scattering from the two points will interfere with each other, resulting in node-like features that were never observed in the concentric rings (Fig. 2). Therefore, we exclude the possibility of electron scattering in the present experiments.

**Discussion of the similarity between the Coulomb blockade model and our explanation**

It is noted that for a quantum dot (QD) consisting of hundreds or thousands of atoms, the semiclassical Coulomb blockade model is applied to treat the QD that is capacitively coupled to the gate electrode, and the charging energy is *e^2^/C*, where *C* is the capacitance of the QD. In our system, the defect state of V_Bi_ is not localized at the position of the defect, but rather is delocalized at a much larger area around the defect than usual (the defect states can be observed even at the sites approximately ~2.3 nm away from the V_Bi_, as shown in Fig. S2). If we treat the defect with delocalized states as a QD with a diameter of approximately 3.0 nm, a charging energy of 0.1 eV derived from an equivalent capacitance of approximately 2.0 aF [38] will be estimated that is close to the peak interval observed in Fig. 1(d). Our explanation and the semiclassical Coulomb blockade model of QD appear to be different at first glance. However, the similar results obtained from these two pictures indicate that their underlying fundamental physics are the same. It is very intriguing that the defect we found is more QD-like, and this phenomenon warrants additional future study.

**First-principles study of intrinsic defects in 2D Sn_2_Bi**

First-principles defect calculations considered various kinds of intrinsic defects in 2D Sn_2_Bi, including vacancies (V_Sn_ and V_Bi_), antisites (Sn_Bi_ and Bi_Sn_) and adatoms (Ad_Sn_ and Ad_Bi_), where Sn_Bi_ denotes Sn on a Bi site. In a perfect 2D lattice of Sn_2_Bi, there are two types Sn sites (Sn^T^ and Sn^C^) located at the boundary and the center of the Bi hexagons, respectively, two types of Bi sites ($\mathrm{Bi}^{T_{4}}$ and $\mathrm{Bi}^{H_{3}}$) located above the T_4_ and H_3_ hollow sites of the Si(111) substrate, respectively, one stable adsorption site for the Sn adatom above a hollow site and one stable adsorption site for the Bi adatom above a Sn-Sn bridge site, as shown in Fig. S3. The most stable sites of the vacancies and antisites are listed below:

- For V_Sn_, the most stable site is on Sn^T^, 1.640 eV lower in energy than that on Sn^C^.
- For V_Bi_, the most stable site is on $\mathrm{Bi}^{H_{3}}$, 0.137 eV lower in energy than that on $\mathrm{Bi}^{T_{4}}$.
- For Sn_Bi_, the most stable site is on $\mathrm{Bi}^{H_{3}}$, 0.009 eV lower in energy than that on $\mathrm{Bi}^{T_{4}}$.
- For Bi_Sn_, the most stable site is on Sn^C^, 0.307 eV lower in energy than that on Sn^T^.

Under the experimental Bi-poor and Sn-rich conditions, the defects with the lowest formation energy are $V_{\mathrm{Bi}}$ and $\mathrm{Sn}_{\mathrm{Bi}}$, for which the electronic structures are presented in Fig. S4.

**Influence of charging induced structural relaxation**

In DFT calculations, structural relaxations were performed without considering charging effects by default. The charging effects modify the optimized atomic structures slightly. For instance, the Sn-Sn distance of the Sn trimer near Bi vacancy decreases from 3.33 Å for *N* = 0 to 3.22 Å for *N* = 4. The charging-induced structural relaxation reduces the defect formation energy of $V_{\mathrm{Bi}}$ by 14.3, 6.2, 28.6, and 51.1meV for *N* = 0, 2, 3, and 4, respectively. The effects of the charging-induced structural relaxation on the electronic structures and defect energy levels are shown in Fig. S9.

**First-principles study of X vacancy in 2D Sn_2_X (X = Sb, As, and P)**

Analogous to the Bi vacancy in 2D Sn_2_Bi, the charging properties of the X vacancy in 2D Sn_2_X (X = Sb, As and P) were also studied. Similar to V_Bi_, the X vacancy introduces four in-gap defect states (D_1,2_ and D_3,4_). These defect energy levels shift upwards when more electrons occupy the defect states, as shown in Fig. S10.

**References**

[34] J. Gou, B. Xia, H. Li, X. Wang, L. Kong, P. Cheng, H. Li, W. Zhang, T. Qian, H. Ding, Y. Xu, W. Duan, K. Wu, L. Chen, Phys. Rev. Lett. **121**, 126801 (2018).

[38] U. Banin, Y. Cao, D. Katz, O. Millo, Nature **400**, 542 (1999).

[39] J. Gou, L. J. Kong, W. B. Li, S. X. Sheng, H. Li, S. Meng, P. Cheng, K. H. Wu, L. Chen, Phys. Chem. Chem. Phys. **20**, 20188 (2018).

[40] I. Horcas, R. Fernandez, J. M. Gomez-Rodriguez, J. Colchero, J. Gomez-Herrero, A. M. Baro, Rev. Sci. Instrum. **78**, 013705 (2007).

[41] G. Kresse, J. Furthmüller, Phys. Rev. B **54**, 11169 (1996).

[42] J. P. Perdew, K. Burke, M. Ernzerhof, Phys. Rev. Lett. **77**, 3865 (1996).

[43] V. Blum, R. Gehrke, F. Hanke, P. Havu, V. Havu, X. Ren, K. Reuter, M. Scheffler, Comput. Phys. Commun. **180**, 2175 (2009).

[44] A. V. Krukau, O. A. Vydrov, A. F. Izmaylov, G. E. Scuseria, J. Chem. Phys. **125**, 224106 (2006).

[45] J. Tersoff, D. R. Hamann, Phys. Rev. B **31**, 805 (1985).

[46] L. Vegard, Zeitschrift für Physik A Hadrons and Nuclei **5**, 17 (1921).

[47] M. Scheffler, Physica B+ C **146**, 176-186 (1987).

[48] Y. Xu, O. T. Hofmann, R. Schlesinger, S. Winkler, J. Frisch, J. Niederhausen, A. Vollmer, S. Blumstengel, F. Henneberger, N. Koch, Phys. Rev. Lett. **111**, 226802 (2013).

[49] F. J. Morin, J. P. Maita, Phys. Rev. **96**, 28 (1954).

[50] B. Krahl-Urban, E. A. Niekisch, H. Wagner, Surf Sci. **64**, 52 (1977).

[51] P. Hahn, J. Clabes, M. Henzler, J. Appl. Phys. **51**, 2079 (1980).

[52] J. Repp, G. Meyer, S. Paavilainen, F. E. Olsson, M. Persson, Phys. Rev. Lett. **95**, 225503 (2005).


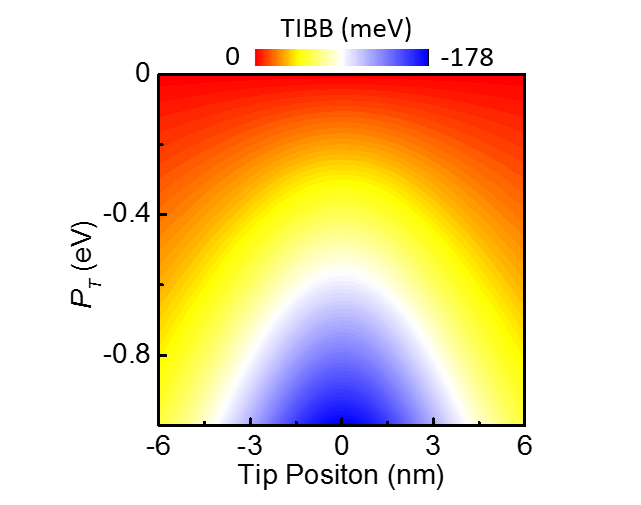


Fig. S1. Calculated TIBB contour as a function of the tip potential energy (*P_T_*) and tip-defect position.


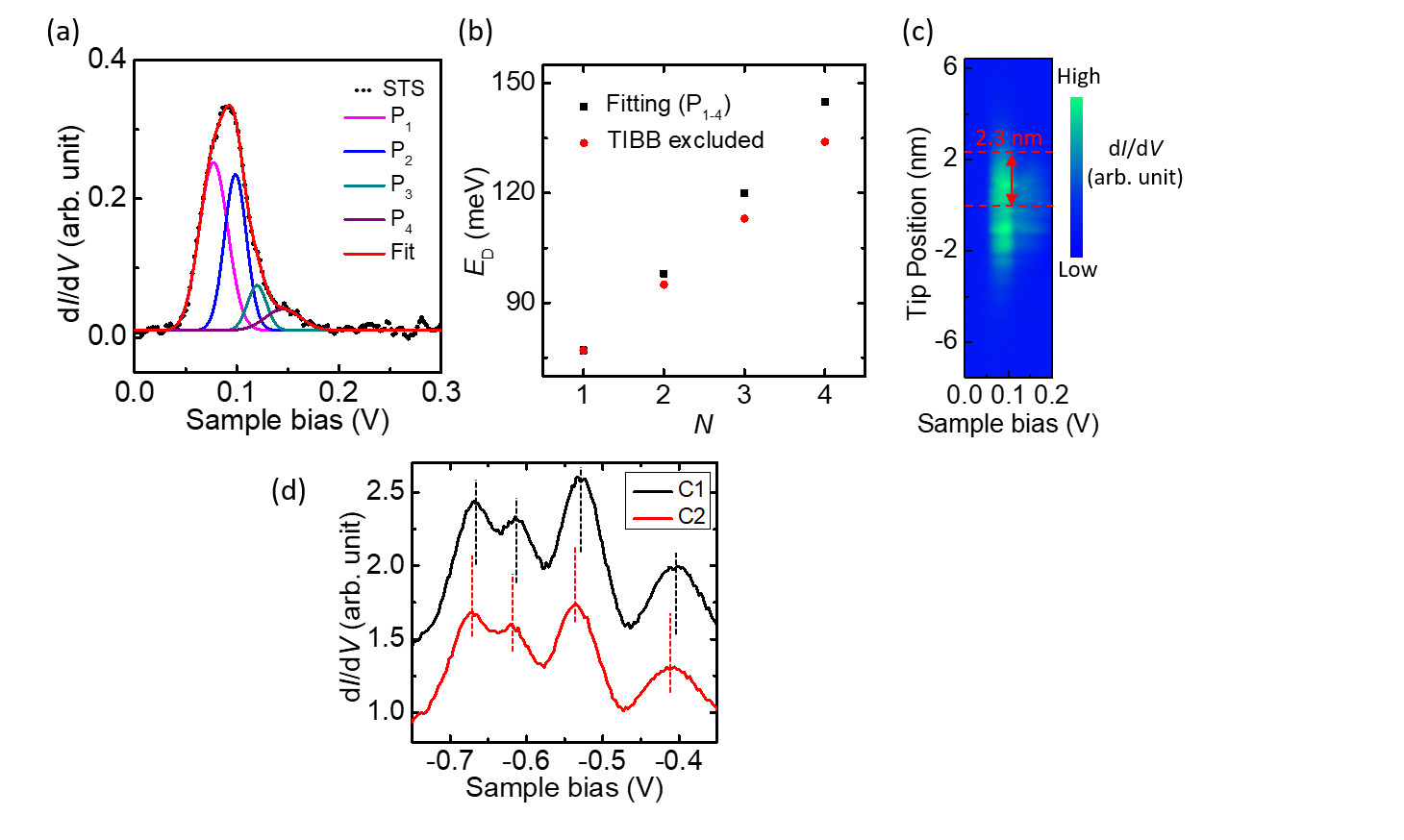


Fig. S2. Closely measurement of in-gap states. (a) STS (setpoint: *V_S_* = -0.2 V, *I* = 84 pA) spectrum measured on a V_Bi_ defect showing the in-gap defect states fitted by four Gaussian curves marked as P_1_-P_4_. (b) The central energy of the four Gaussian peaks (black) and TIBB excluded results (red). (c) d*I*/d*V* line maps taken cross over a Bi vacancy (initiate setpoint: *V_S_* = 0.9 V, *I* = 98 pA). (d) d*I*/d*V* curve measured above a Bi vacancy at different heights (initiate setpoint: C1, *V_S_* = -1.0 V, *I* = 400 pA; C2, *V_S_* = -1.3 V, *I* = 400 pA).


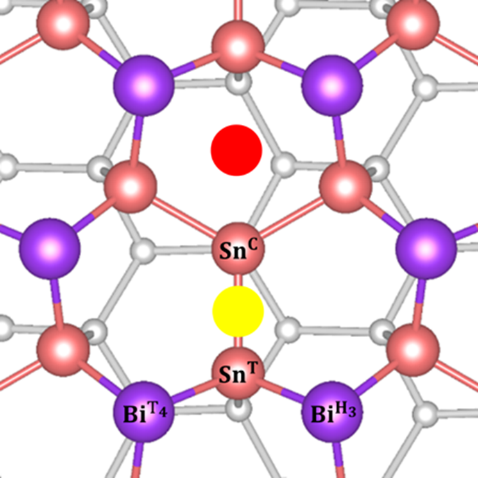


Fig. S3. Different atomic sites in 2D Sn_2_Bi. There are two types of Sn sites (Sn^T^ and Sn^C^), two types Bi sites ($\mathrm{Bi}^{T_{4}}$ and $\mathrm{Bi}^{H_{3}}$), one stable adsorption site for Sn adatom (red ball) and one for Bi adatom (yellow ball).


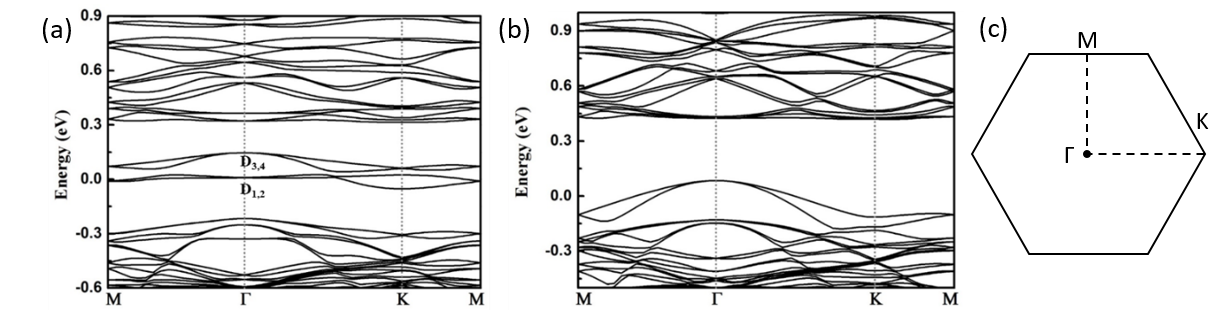


Fig. S4. Electronic structures of $V_{\mathrm{Bi}}$ and $\mathrm{Sn}_{\mathrm{Bi}}$ in 2D Sn_2_Bi. Band structures of 2D Sn_2_Bi including (a) one $V_{\mathrm{Bi}}$ or (b) one $\mathrm{Sn}_{\mathrm{Bi}}$ per 6×6 surface supercell. (c) The Brillouin zone and the definition of high-symmetry points (Γ, M, K) mentioned in our manuscript are illustrated.


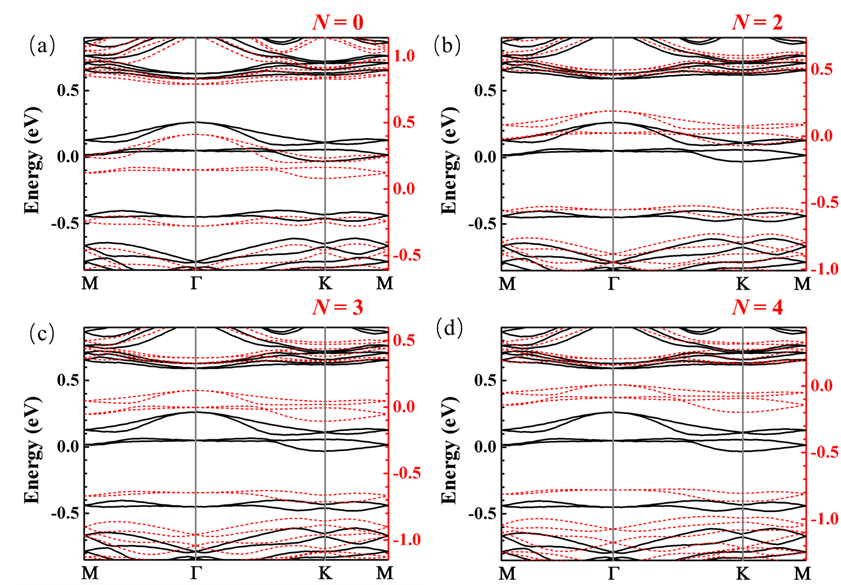


Fig. S5. Test calculations by the DFT-HSE06 method. DFT-HSE06 band structures of the 6×6 surface supercell with one Bi vacancy for different defect charging states (red dotted lines): (a) *N* = 0, (b) *N* = 2, (c) *N* = 3, and (d) *N* = 4 are compared to the corresponding results for *N* = 1 (black solid lines). The DFT-HSE06 calculations indicate that the defect states always stay within the band gap for different charging states from *N* = 0 to *N* = 4. The same conclusion was obtained by DFT-PBE calculations.


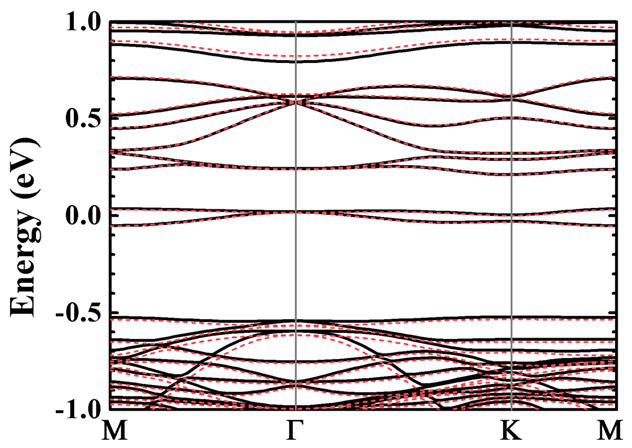


Fig. S6. Tests of the theoretical methods for charging simulation. Band structures of the 8×8 surface supercell with one Bi vacancy and defect charging states *N* = 2 are obtained by two methods: (i) the compensating charge was introduced by a homogeneous charge background (black solid lines); (ii) the virtual crystal approximation was applied (red dotted lines). The two methods give essentially the same results.


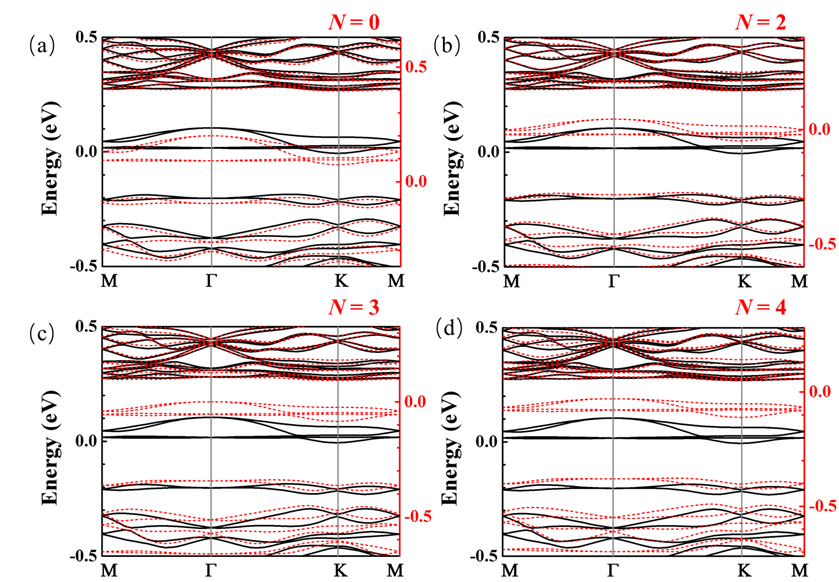


Fig. S7. Electronic structures of Bi vacancy in 2D Sn_2_Bi. Band structures of the 8×8 surface supercell with one Bi vacancy for different defect charging states (red dotted lines): (a) *N* = 0, (b) *N* = 2, (c) *N* = 3, and (d) *N* = 4 are compared to the corresponding results for *N* = 1 (black solid lines).


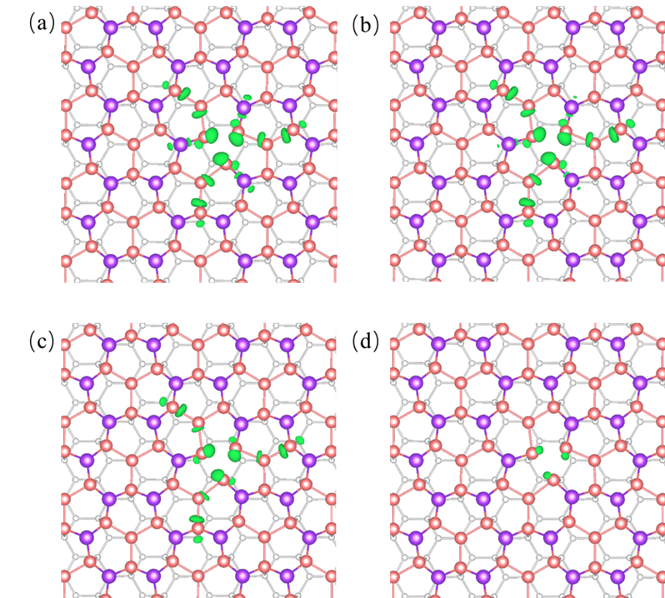


Fig. S8. Real-space charge redistribution induced by successive charging. Charge density differences between charging states *N* and *N*-1 are calculated, and their isosurfaces are presented for (a) *N* = 1, (b) *N* = 2, (c) *N* = 3, and (d) *N* = 4. The isosurface value is 3.5×10^-4^ *e*/bohr^3^.

**
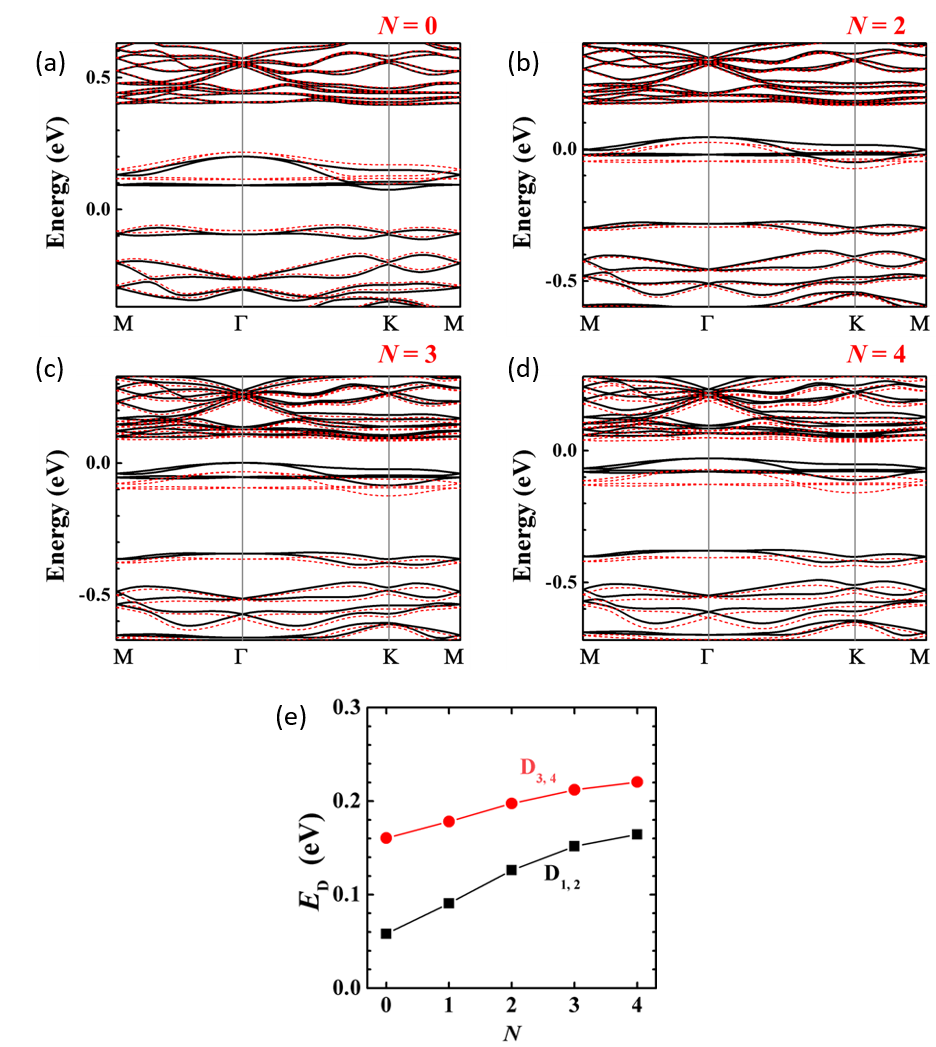
**

Fig. S9. Influence of charging-induced structural relaxation on electronic structure of Bi vacancy in 2D Sn_2_Bi. Band structures of the 8×8 surface supercell with one Bi vacancy for different defect charging states: (a) *N* = 0, (b) *N* = 2, (c) *N* = 3, and (d) *N* = 4, excluding (black solid lines) and (red dotted lines) including charging-induced structural relaxation. (e) Defect energy levels of D_1,2_ and D_3,4_ (referenced to the VBM of Sn_2_Bi) for different defect charging states. *N* denotes the occupation of the in-gap defect states.

**
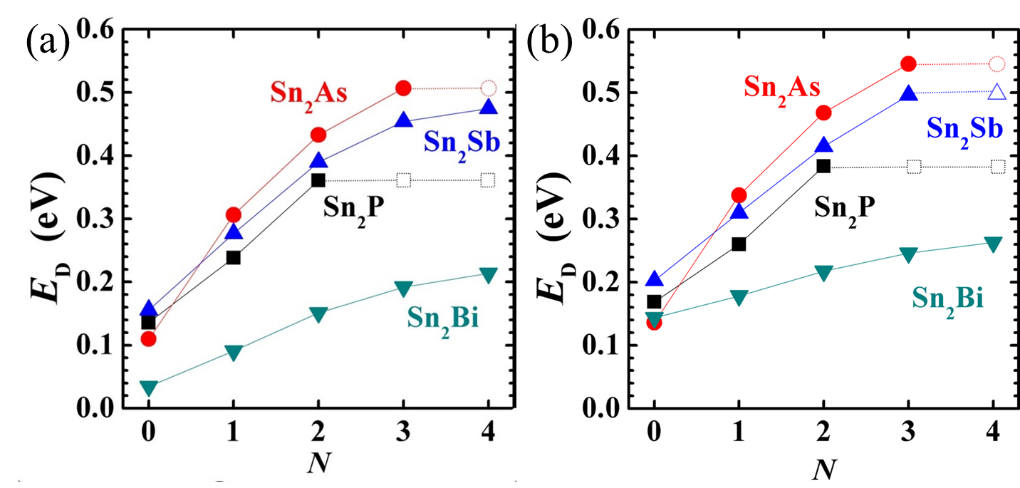
**

Fig. S10. Calculated defect energy levels of X vacancy in 2D Sn_2_X (X = Bi, Sb, As, and P). The defect energy levels of (a) D_1,2_ and (b) D_3,4_ (referenced to the VBM of Sn_2_X) for different defect charging states *N*. Solid dots mean that all of the defect states are within the band gap, whereas open dots mean that some of the defect states are shifted outside the band gap and electrons are doped into the bulk conduction bands. Generally, defect energy levels shift upwards with increasing *N*. When varying X from Bi, Sb, As to P, the upward shift becomes increasingly pronounced. As a result, the defect states cannot be fully occupied and stay within the band gap in Sn_2_As and Sn_2_P.
